# Generation and assessment of cytokine-induced killer cells for the treatment of colorectal cancer liver metastases

**DOI:** 10.1007/s00262-023-03591-4

**Published:** 2024-01-17

**Authors:** Celine Man Ying Li, Yoko Tomita, Bimala Dhakal, Teresa Tin, Runhao Li, Josephine A. Wright, Laura Vrbanac, Susan L. Woods, Paul Drew, Timothy Price, Eric Smith, Guy J. Maddern, Kevin Fenix

**Affiliations:** 1 Discipline of Surgery, Adelaide Medical School, The University of Adelaide, Adelaide, 5005 Australia; 2 The Basil Hetzel Institute for Translational Health Research, The Queen Elizabeth Hospital, Adelaide, 5011 Australia; 3 Medical Oncology, The Queen Elizabeth Hospital, The University of Adelaide, Adelaide, 5011 Australia; 4 Precision Medicine, South Australian Health and Medical Research Institute, Adelaide, 5005 Australia; 5 Department of Medical Specialties, Adelaide Medical School, The University of Adelaide, Adelaide, 5005 Australia

**Keywords:** Cytokine-induced killer cells, Colorectal cancer, Liver metastases, Patient-derived tumour organoids

## Abstract

Colorectal cancer (CRC) is the second leading cause of cancer-related death worldwide. Cytokine-induced killer (CIK) cells are an adoptive immunotherapy reported to have strong anti-tumour activity across a range of cancers. They are a heterogeneous mix of lymphoid cells generated by culturing human peripheral blood mononuclear cells with cytokines and monoclonal antibodies in vitro. In this study, we investigated the yield and function of CIK cells generated from patients with CRC liver metastases. We first showed that CIK cells generated in serum free medium X-VIVO 15 were comparable to those from RPMI medium with 10% FBS in terms of the number and percentages of the main subsets of cells in the CIK culture, and the intracellular levels of granzyme B and perforin, and the pro-inflammatory cytokines IL-2, IFN-γ and TNF-α. The CIK cells were cytotoxic to CRC cell lines grown in 2D cultures or as spheroids, and against autologous patient-derived tumour organoids. Donor attributes such as age, sex, or prior chemotherapy exposure had no significant impact on CIK cell numbers or function. These results suggest that functional CIK cells can be generated from patients with CRC liver metastatic disease, and support further investigations into the therapeutic application of autologous CIK cells in the management of patients with CRC liver metastases.

**Supplementary Information:**

The online version contains supplementary material available at 10.1007/s00262-023-03591-4.

## Introduction

Colorectal cancer (CRC) is the second leading cause of cancer-related death worldwide [1]. The 5-year survival rate of CRC is 75%, but decreases to 30% with metastatic disease [2]. Currently, 25–70% of patients develop colorectal cancer liver metastases (CRLM) [3, 4]. Treatment options for CRLM patients include surgical resection and chemotherapy, but only a small proportion of CRLM patients are eligible for surgical resection and chemotherapy resistance commonly develops [5]. Thus, there is still a great need for more effective therapies for late-stage CRCs such as CRLM [6].

Cytokine-induced killer (CIK) cell therapy is a cellular adoptive immunotherapy first described in the 1990s [7]. CIK cells are cultured from peripheral blood mononuclear cells (PBMCs) to generate a heterogeneous mix of immune effector cells. Well-described subpopulations include conventional T cells (CD3 + CD56-), natural killer (NK)-like T cells (CD3 + CD56 +), and NK cells (CD3-CD56 +). The expression of both T cell and NK cell receptors by CIK cells allows for both major histocompatibility complex (MHC)-dependent and independent tumour recognition. CIK cells have been reported to have strong anti-tumour activity across a range of cancers, including solid tumours [8]. CIK cells can induce tumour cytotoxicity by release of cytolytic granules and expression of death ligands (FASL and TRAIL). They also mount a type-1 inflammatory response by releasing interleukin (IL)-2, interferon (IFN)-γ and tumour necrosis factor (TNF)-α [7, 9]. Meta-analyses of CRC clinical trials for CIK cell therapy, mostly conducted in China, have shown significant improvement in patient outcomes, including overall and progression-free survival [10, 11].

An advantage of CIK cell therapy is its relative ease of production and inexpensive material costs. Briefly, PBMCs are cultured for up to 28 days in the presence of IFN-γ, anti-CD3, and IL-2 [8]. Quality control of the culture product is currently based on counting viable CD3 + CD56- and CD3 + CD56 + cells by flow cytometry. It is generally considered that CIK cells for patient infusion should contain about 90% CD3 + T cells with expansion of T cells co-expressing CD56. Most laboratory and some clinical studies generated CIK cells in media supplemented with foetal bovine serum (FBS) [12–15]. However, animal-derived serum components have the potential to increase the risk of adverse events [16] and thus are not suitable for use in clinical good manufacturing practice (GMP) protocols.

In this study, we confirm that X-VIVO 15 is a suitable GMP compliant T cell culture specific serum free medium to expand CIK cells from PBMC from healthy or CRLM donors. The CIK cell numbers and functionality, including cytotoxicity to tumour cells, were similar between the two groups. Patient characteristics such as age, sex, and prior chemotherapy did not significantly impact on CIK cell numbers or function. This study supports the application of autologous CIK cell therapy as a potential treatment for patients with CRLM.

## Materials and methods

### Study group

Healthy donors or patients with CRLM were consented at The Queen Elizabeth Hospital (TQEH, Woodville, South Australia). Donor data are recorded in Table 1 and Supplementary Tables 1 and 2. Donor Patient IDs were generated by the research group and cannot be used to identify patients external to the group. This study was approved by the Human Research Ethics Committee of the Central Adelaide Local Health Network under protocol number HREC/14/TQEHLMH/164.

**Table 1:** Characteristics of healthy donors and patients with liver colorectal cancer metastases

Characteristics	CRLM patients	Healthy donors
Total number (*n*)	17	8
Gender (Male/Female)	13/4	6/2
Median age	58	35
*Cancer origins*		
Caecum (*n*)	3	–
Rectum (*n*)	4	
Left/right colon (*n*)	10	
*Chemotherapy exposure*		
Before PBMC collection (*n*)	11	–

### CIK cell generation

The PBMCs were isolated from donor venous blood using Ficoll-Paque (Bio-Strategy, USA) density gradient centrifugation as previously described [17]. The patient blood was collected just before curative intent liver resection for CRLM. Cells were cultured in a 24- or 12-well tissue culture grade plates. Complete RPMI is defined as RPMI 1640 medium (Life Technologies, USA) containing 10% FBS (Sigma-Aldrich, USA), 1X L-Glutamine (Gibco, USA), and 200 U/mL penicillin and 200 µg/mL streptomycin (pen-strep) (Life Technologies, USA). Serum-free media, X-VIVO 15 (LONZA, Switzerland), TexMACS (Milltenyi Biotec, USA), and CTS OpTmizer (Gibco, USA) were supplemented with pen-strep. Briefly, isolated PBMCs were seeded at 2 × 10^6^ cells/mL in media containing 1000 U/mL of interferon gamma (IFN-γ) (Miltenyi Biotec, USA). After 24 h, 0.05 µg/mL of anti-CD3 (Miltenyi Biotec, USA) and 300 IU of interleukin (IL)-2 (Miltenyi Biotec, USA) were added. Three days post anti-CD3 stimulation, culture media were topped up with 500 µL of media containing 300 IU of IL-2. The cells were split every 3–4 days and seeded at density of 1 × 10^6^ cells/mL with the addition of 300 IU IL-2 for up to 21 days from isolation of the PBMCs.

### Flow cytometry

Cells were stained with BD Horizon Fixable Viability Stain 780 (FVS780) (Biolegend, USA). Cells were treated with 50 µL of Fc block (BD Biosciences, USA), and then stained with anti-human CD3 V510, anti-human CD56 PeCy-7, anti-human CD4 FITC, anti-human CD8 647, anti-human CD226 PE, and anti-human CD314 V421 (Biolegend, USA) prepared in FACS buffer (2% FBS, 0.05% sodium azide and 1 mM EDTA into sterile 1X PBS) for 30 min at 4 °C. After washing with FACS buffer, the fluorescence data were acquired using a FACS Canto II flow cytometer (BD Biosciences, USA). For measurement of intracellular markers, cells were stimulated and cultured with 1X Cytokine Activation Cocktail (BD Biosciences, USA) for 5 h. They were then permeabilised with Intracellular Fixation Buffer (BD Biosciences, USA) for 20 min and washed with permeabilization buffer/wash buffer (BD Biosciences, USA). They were stained with anti-human granzyme B BV421 and anti-human perforin PerCP5.5 for intracellular cytotoxicity markers or with anti-human tumour-necrosis factor (TNF)-α PerCP5.5 and anti-human interferon IFN-γ BV421 and anti-human IL-2 PE for pro-inflammatory cytokines. Cells were resuspended in 150–200 µL of FACS buffer and the fluorescence data was acquired in the FACS Canto II Flow Cytometry system. The data were analysed by FlowJo v10.8.1 software (BD Biosciences, USA).

### Cryopreservation

The CIK cells were centrifuged and resuspended in FBS with 10% DMSO at a concentration of 1 × 10^6^ cells/mL. Aliquots in cryovials were frozen using a Mr. Frosty Freezing Container (Thermo Fisher Scientific, USA) at -80 °C overnight before being stored long term in liquid nitrogen. To thaw out the cells, the vial was defrosted in a 37 °C water bath. Immediately after thawing, the cells were gently transferred in a drop-wise fashion into a 15 mL falcon tube containing 1 mL pre-warmed culture media.

### Cell lines

The CRC cell lines (HT-29, SW620, SW480, COLO 205) were obtained from the American Type Culture Collection (ATCC, USA). HT29 and COLO 205 were maintained in RPMI supplemented with 10% heat-inactivated FBS, 200 U/mL penicillin, 200 µg/mL streptomycin and 200 mM GlutaMAX Supplement (Life Technologies, USA). SW620 and SW480 were maintained in DMEM (Life Technologies, USA) with the same supplementation as RPMI. Cells were incubated at 37 °C with 5% CO_2_.

### Patient-derived tumour organoids

To establish patient-derived tumour organoids (PDTOs), tumour samples were collected from patients undergoing liver resection for CRLM disease and cultured as described previously [18]. Briefly, CRLM tissues were minced and digested in organoid digestion buffer containing 2.5% of FBS, 75 U/ml Collagenase Type IV (Gibco, USA), 125 µg/ml dispase (Gibco, USA), 20 µg/mL hyaluronidase (Sigma-Aldrich, USA) and 10 µM Y27632 (Sigma-Aldrich, USA) in advanced DMEM media (Gibco, USA) for 30–60 min in a water bath at 37 °C. After the tissues were completely digested, single cells were obtained and pelleted by centrifugation. If pellets were contaminated with red blood cells, red blood cell lysis was performed using ACK lysis buffer (Gibco, USA). The pellet was resuspended in a volume of pre-thawed phenol-red free Matrigel (Life Technologies, USA) at 4 °C depending on the density of the cells. Single cells were embedded in the 50 µL Matrigel domes and were cultured in 5–6% low oxygen conditions at 37 °C incubator for 30 min. Matrigel domes were then topped with CRC media containing advanced DMEM media, 10 mM HEPES, 1X GlutaMAX Supplement, 1X antibiotic–antimycotic, 10 mg/L Gentamicin, 2X B27 (all from Life Technologies, USA), with the addition of 500 nM A 83–01 (Tocris Bioscience, Bristol, UK), 50 ng/mL hEGF, 1 nM Gastrin, 1 mM N-acetyl-L cyst, 5 µM SB202190, 10 µM SB431542, 10 µM Y27632 (all from Sigma-Aldrich, USA). Organoids were maintained in the same media at 550 µL, and were passaged every one to two weeks, or when they reached 100–200 mm in diameter, by digestion with TrypLE (Life Technologies, USA) at 37 °C. Organoids were considered ready for use after two weeks from initial establishment.

### PDTO cytotoxicity assay

The PDTO cytotoxicity assay was performed as previously described [19]. PDTOs were cultured in a 24-well plate. First, a single well was harvested for cell number estimation. Briefly, PDTOs were washed with ice-cold 1X PBS twice and digested into single cells using 5 mL of TrypLE supplemented with 10 µM Y27632 at 37 °C for less than 15 min. Then 50 µL of FBS was added to stop digestion before the cell count was performed. The rest of the PDTOs were then harvested and labelled with a 1 in 4000 dilution of CellTrace™ Violet (Invitrogen, USA) for 20 min in the dark at room temperature. The PDTOs were seeded into a 96-well flat bottom plate at an equivalent of 1 × 10^5^ cells per well. CRC media supplemented with 10 µg/mL DNase and 300 IU IL-2 was then added into each well. The CIK cells were resuspended in CRC media and co-cultured with PDTOs at effector to target (E:T) ratios of 10:1, 5:1 or 1:1 for 24 h at 37 °C. After 24 h of incubation, supernatants were collected and 100 µL of TrypLE were added into each well for single-cell dissociation followed by viability staining. Samples were then acquired using a Cytek Aurora spectral flow cytometer (Cytek Biosciences) with volumetric counting. For quantification of the percentage of specific lysis, the following formula was used:

$${\text{Specific lysis}}\left( \% \right) = \left( {\left( {{\text{TC}} - {\text{TE}}} \right)/{\text{TC}}} \right) \times {1}00$$

where TC indicates the cell count of live labelled target cells in the control well (target cells alone), and TE indicates the cell count for live labelled target cells in the treatment well (target cells + effector cells) [20].

### CRC cell line cytotoxicity assay

HT-29, COLO 205, SW480 and SW620 cells, stained with CellTrace™ Violet, were seeded at 1 × 10^4^ cells per well in 96-well flat bottom plates and incubated for 24 h at 37 °C. Detached non-viable cells were removed by washing with Dulbecco’s phosphate buffered saline (DPBS) (Life Technologies, USA), CIK cells at 10:1, 5:1 or 1:1 E:T ratio were then added co-cultured with the remaining attached cells for 24 h. All cells were then harvested by pooling non-adherent cells and the adherent cells were detached by incubation with trypsin at 37 °C for 4 min. The detached adherent cells were then washed three times with DPBS and pooled with non-adherent cells. Samples were labelled with BD Horizon FVS780 viability dye and measured using a Cytek Aurora spectral flow cytometer. Specific lysis was calculated using the same formula as the PDTO cytotoxicity assay.

### 3D live cell imaging cytotoxicity assay

Caspase 3/7 activation in response to CIK cells was measured in HT-29 CRC spheroids [21]. HT-29 cells were seeded in a 96-well round bottom ultra-low attachment plate (Corning, New York, NY, USA) at 1 × 10^5^ cells per well and incubated for 72 h to form 3D spheroids. The CIK cells were added to the spheroids to a final ratio of 10:1, 5:1 or 1:1 ratio together with 1 µM CellEvent Caspase-3/7 Green Detection Reagent (Thermo Fisher Scientific, Waltham, MA, USA). The activation of caspase 3/7 was monitored for the following 24 h of incubation and the results were captured and analysed using an Incucyte S3 Live-Cell Analysis System (Sartorius, Germany).

### Statistical analysis

Statistical analyses performed are described in the figure legends. All data were analysed using GraphPad Prism Version 9 (GraphPad Software, USA).

## Results

### Impact of serum-free media in the generation and functionality of CIK cells

**Fig. 1:**
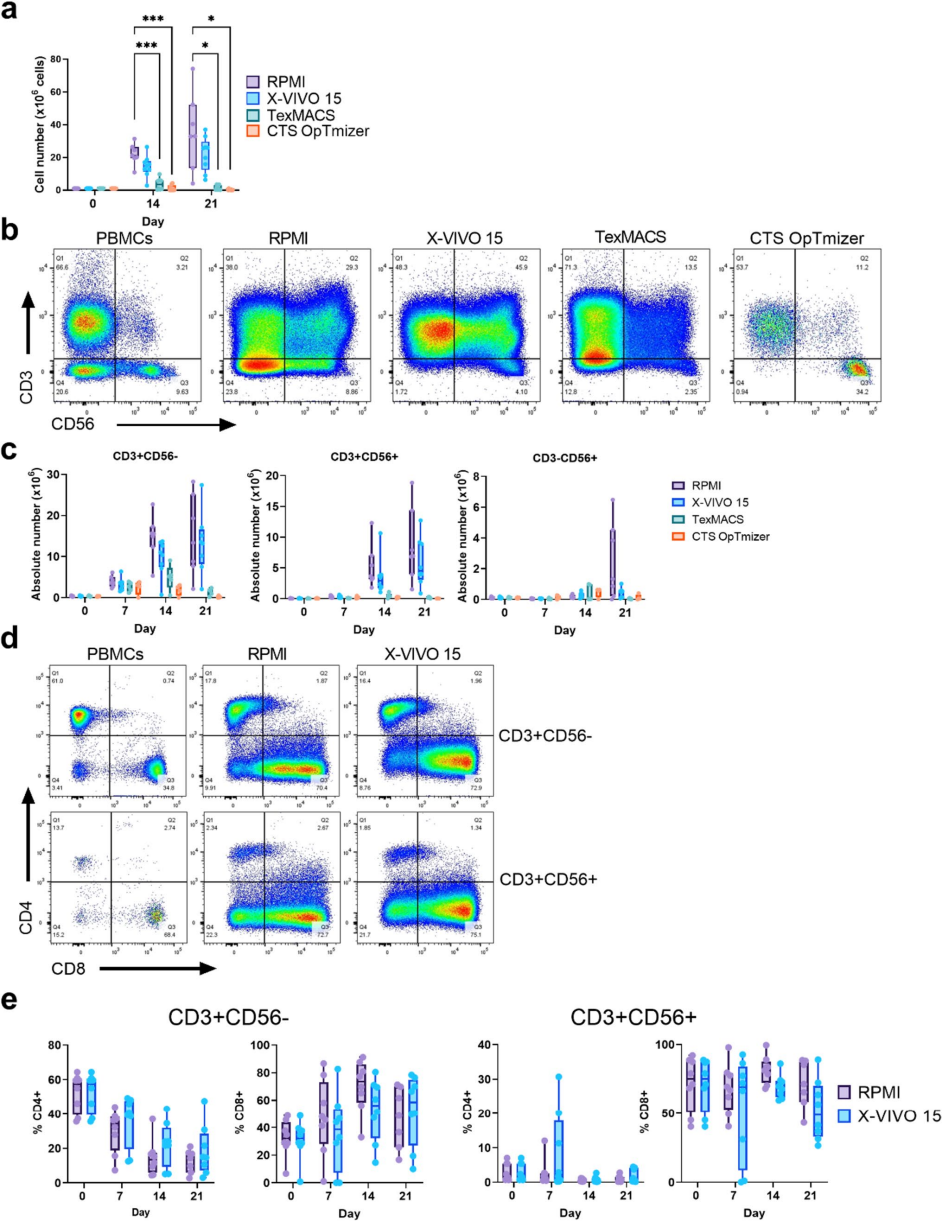
**Impact of serum-free media on CIK cell production.** CIK cells were generated from healthy donor PBMCs using three serum-free media (X-VIVO 15, TexMACS or CTS OpTmizer) or RPMI supplemented with 10% FBS. **a** Total cell counts on Day 0, 14, and 21 of culture. **b** Representative flow cytometry plots of PBMCs and CIK cells at day 21 post-culture and **c** absolute number counts of T cells (CD3 + CD56-), NK-like T cells (CD3 + CD56 +), NK cells (CD3-CD56 +) at different culture time-points. **d** Representative flow cytometry plots showing CD4 + and CD8 + subsets within the CD3 + CD56- and CD3 + CD56 + subpopulations at day 21 of culture in RPMI or X-VIVO 15 media. **e** Quantitation of CD4 and CD8 single positive subpopulations within CD3 + CD56- cells and CD3 + CD56 + cells between RPMI and X-VIVO 15 media. Box plots represent the median with each point representing an individual donor. *p ≤ 0.05, ***p ≤ 0.005. Two-way ANOVA with multiple comparisons test were performed to compare the different media

We first compared CIK cells from the PBMC of healthy donors generated using three GMP-grade serum free media (X-VIVO 15, TexMACS and CTS OpTmizer) or complete RPMI (supplemented with 10% FBS). The only one of these which supported similar expansion of total cells compared to complete RPMI was X-VIVO 15 (Fig. 1a and Supp. Fig. 1). Expansion of the CD3 + CD56 + cell subpopulation was observed in all media, however, only X-VIVO 15 had numbers and percentages close to those in complete RPMI (Fig. 1b). As expected, CD3 + CD56- cells were the largest subpopulation in all media tested. X-VIVO 15 supported expansions of CD3 + CD56- cells and CD3 + CD56 + cells to 13.36 (2.08–27.42) and 4.54 (0.86–12.7) × 10^6^, respectively (Fig. 1c and Supp. Table 3). Mature CD3 + CD56- and CD3 + CD56 + populations can be further as CD4 + T helper cells or CD8 + cytotoxic T lymphocytes (CTLs) [22]. CIK cells have been shown to consist mostly of CD8 + cells [23]. CIK cells grown in complete RPMI led to CTL expansion that did not differ from X-VIVO 15 with median of 48.7 (16.6–71.4) and 71.4 (43.3–88.7) × 10^6^, respectively (Fig. 1d, e and Supp. Table 4).

**Fig. 2:**
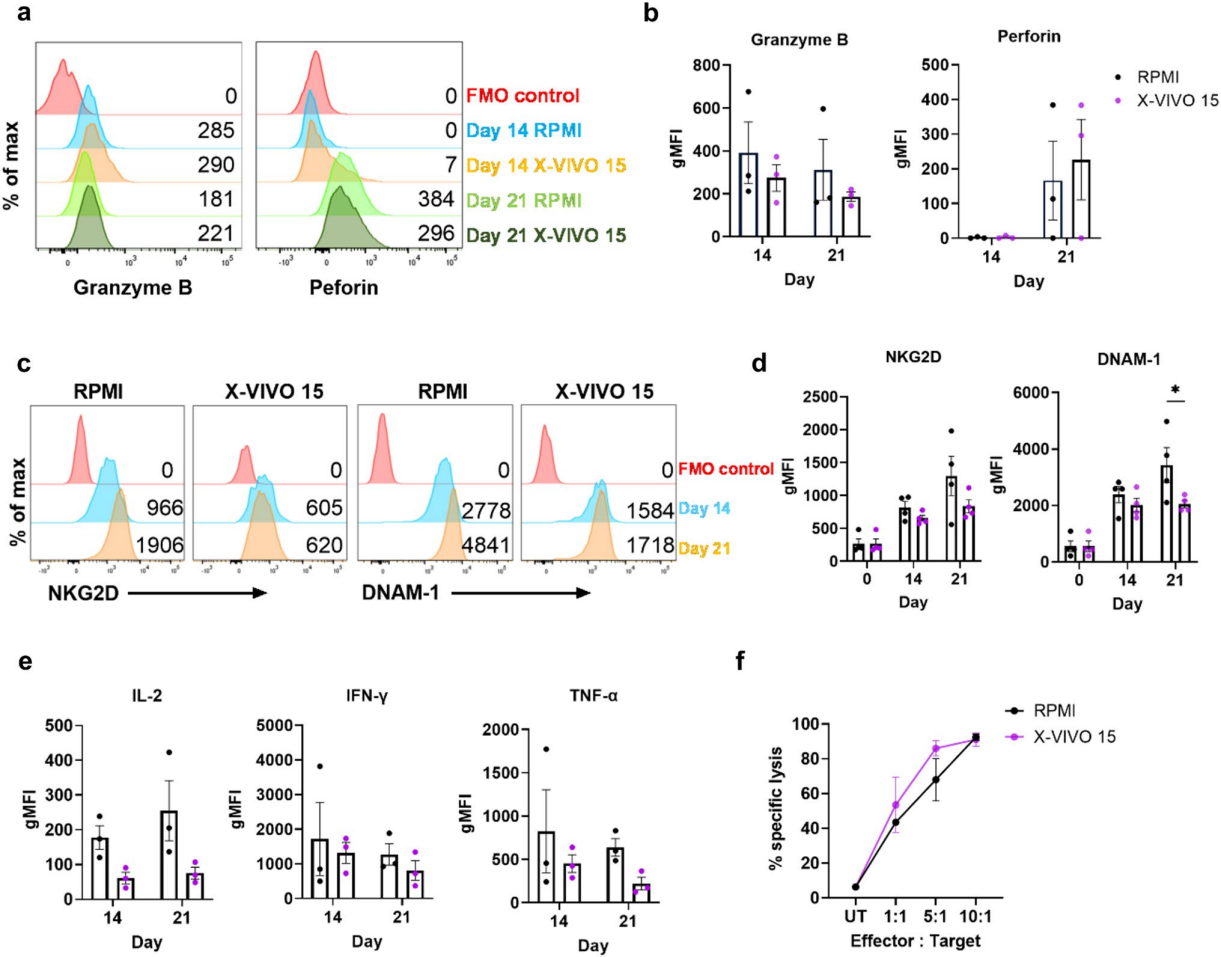
**Properties of CIK cells generated in X-VIVO 15 or RPMI.** Flow cytometric analysis of CIK cells identified by live CD8 + CD3 + CD56 + cells. **a** Representative histograms and **b** the geometric mean fluorescence intensity (gMFI) of intracellular granzyme B and perforin (*n* = 3). **c** Representative histograms and **d** gMFI of NKG2D and DNAM-1 expression (*n* = 4). **e** The gMFI for intracellular IL-2, IFN-γ and TNF-α. (*n* = 3). **f** 2D cytotoxicity assay with the HT-29 CRC cell line as target cells and CIK cells at 10:1, 5:1, 1:1 effector to target (E:T) ratios. Target cells only were used as untreated (UT) controls (*n* = 3). *p ≤ 0.05. Two-way ANOVA with multiple comparisons test between the mean of RPMI and X-VIVO 15 media

Next, we determined if CIK cells in X-VIVO 15 had similar capacity against cancer cells as those generated in complete RPMI. First, CIK cells are mostly CD8 + CD3 + CD56- and CD8 + CD3 + CD56 + cells, which mediate cell death by the release of cytolytic granules including perforin and granzymes during immunological synapse with their targets [24]. The intracellular expression of granzyme B and perforin in CD8 + CD3 + CD56 + cells did not differ between CIK cells generated in X-VIVO 15 or RPMI, consistent with these cells having similar cytolytic capacities, with granzyme B positive expression were 14.86% (mean ± SD 23.78) at day 14 and 7.30% (mean ± SD 12.56) at day 21 in RPMI media, and 13.21% (mean ± SD 10.28) at day 14, and 1.18% (mean ± SD 0.58) at day 21 in X-VIVO 15 media. While perforin positive expressions were 0.78% (mean ± SD 1.09) at day 14 and 4.44% (mean ± SD 6.41) at day 21 in RPMI, and 0.96% (mean ± SD 0.55) at day 14, and 11.21% (mean ± SD 7.024) at day 21 (Fig. 2a, b) (Supp. Table. 5). Similar findings were observed for CD8 + CD3 + CD56- cells (Supp. Fig. 2a).

CIK cells can recognise cancer cells by MHC-independent mechanisms through NK cell receptors [23]. Expression of NKG2D and DNAM-1, the main NK cell-activating receptors that lead to granule exocytosis, cytokine secretion and cellular cytotoxicity in CIK cells [25]. Expression of both receptors in the CD8 + CD3 + CD56 + cells increased significantly during culture (Fig. 2c, d). Further, the expressions of NKG2D and DNAM-1 were similar in both CD4 + and CD8 + subpopulations of the CD3 + cells and the CD3 + CD56 + cells grown in the two media. However, DNAM-1 expression was greater in CIK cells grown in RPMI compared to those grown in X-VIVO 15 at day-21 post-culture (Fig. 2d) (Supp. Fig. 2b).

We then determined if CIK cells grown in X-VIVO 15 produced IL-2, IFN-γ and TNF-α, type-1 cytokines that are commonly associated with CTLs and are expressed by CIK cells [26]. Flow cytometric intracellular staining for these cytokines in the CD3 + CD56 + cells, showed less expression in cells grown in X-VIVO 15 than RPMI (Fig. 2e and Supp. Fig. 3). Finally, we examined the cytotoxic capacity of these cells against HT-29 CRC cell lines grown as 2D monolayers. There was a dose specific cytotoxic response against target cells, with no difference observed between cells grown in X-VIVO 15 or RPMI (Fig. 2f).

Recently it was reported that CIK cells can be cryopreserved with no significant loss of in vitro or in vivo cytotoxic potency [27]. Since treatment regimens for CIK therapy include multiple rounds of CIK infusion over many months [5, 28], the ability to be able to use frozen aliquots from one large batch of CIK cells might be of practical benefit. We thus investigated if cryopreservation storage time affects the cytotoxic capacity of CIK cells. Comparing cells stored in liquid nitrogen either for 1–4 or 6–12 months showed that the longer-term cryopreservation can significantly reduce cytotoxic activity (Supp. Fig. 4), suggesting that cryopreservation, while useful, should be for shorter term storage. Together, these data suggest that X-VIVO 15 is a suitable serum free medium for the generation of CIK cells.

### Characterisation of CIK cells derived from CRLM donors

**Fig. 3:**
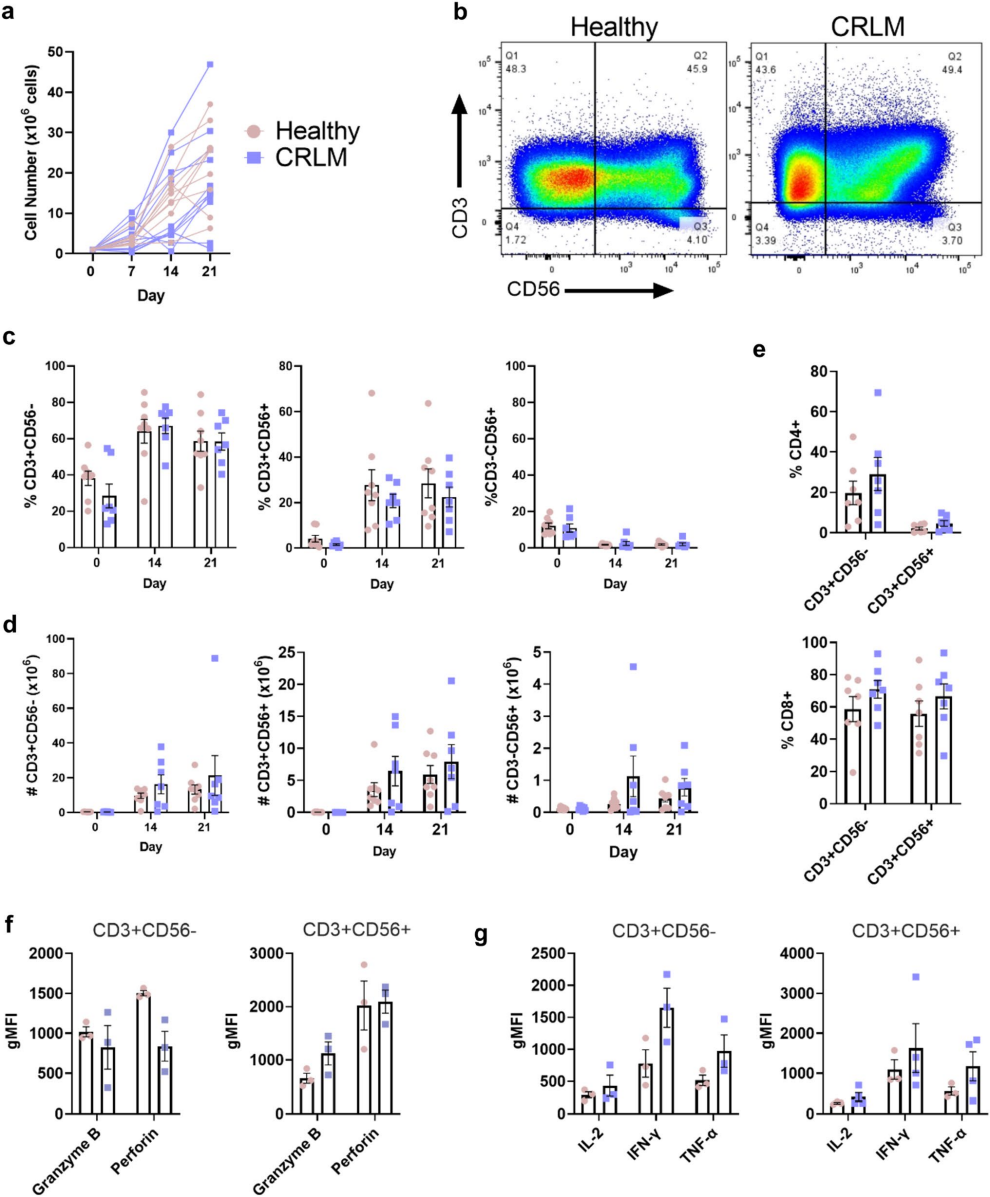
**Comparison of CIK cells from CRLM and healthy donors.** CIK cell expansion of PBMC from CRLM or healthy donors grown in X-VIVO 15 media. **a** Total cell counts on days 0, 7, 14, and 21 of culture. **b** Representative flow cytometry plots in PBMCs and CIK cells at day 21 of culture and **c** percentage and **d** absolute number counts of T cells (CD3 + CD56-), NK-like T cells (CD3 + CD56 +), NK cells (CD3-CD56 +) at different cell culture time points. **e** The percentage of CD4 + and CD8 + subsets within the CD3 + CD56- and CD3 + CD56 + subpopulations at day 21 of culture. **f** The gMFI for intracellular granzyme B and perforin and **g** IL-2, IFN-γ and TNF-α in CD8 + CD3 + CD56- and CD8 + CD3 + CD56 + CIK cells. Data are shown as mean ± SEM with each point representing an individual donor

We recently reported that past clinical studies on CIK cell therapy for CRC were generated from autologous patient-derived PBMCs [11]. Thus, we investigated if PBMCs from CRLM donors have similar capacity to produce CIK cells as healthy donors. In total, 17 CRLM donors were recruited for this study (Table 1). PBMCs from both CRLM and healthy donors could generate CIK cells, although the number of cells expanded varied greatly, with some PBMCs from both healthy and CRLM donors failing to expand (Fig. 3a). In the samples that successfully expanded, the numbers and percentages of CD3-CD56 + , CD3 + CD56-, and CD3 + CD56 + cells did not differ significantly between CRLM and healthy donors (Fig. 3b–d). The percentages of CD4 + and CD8 + subpopulations in the CD3 + CD56- and CD3 + CD56 + cells were not significantly different between the donor groups. As expected, there were significantly more CD8 + than CD4 + cells (Fig. 3e). We compared the functional capacity of the CD3 + CD56- and CD3 + CD56 + subpopulations of patients and controls and found similar expression of cytolytic molecules: granzyme B and perforin (Fig. 3f) and type-1 cytokines, IL-2, IFN-γ and TNF-α (Fig. 3g). These results indicate that PBMC from donors with CRLM can produce CIK cells in equivalent numbers and with equivalent expression of functional molecules, consistent with the possibility of them being functionally equivalent.

**Fig. 4:**
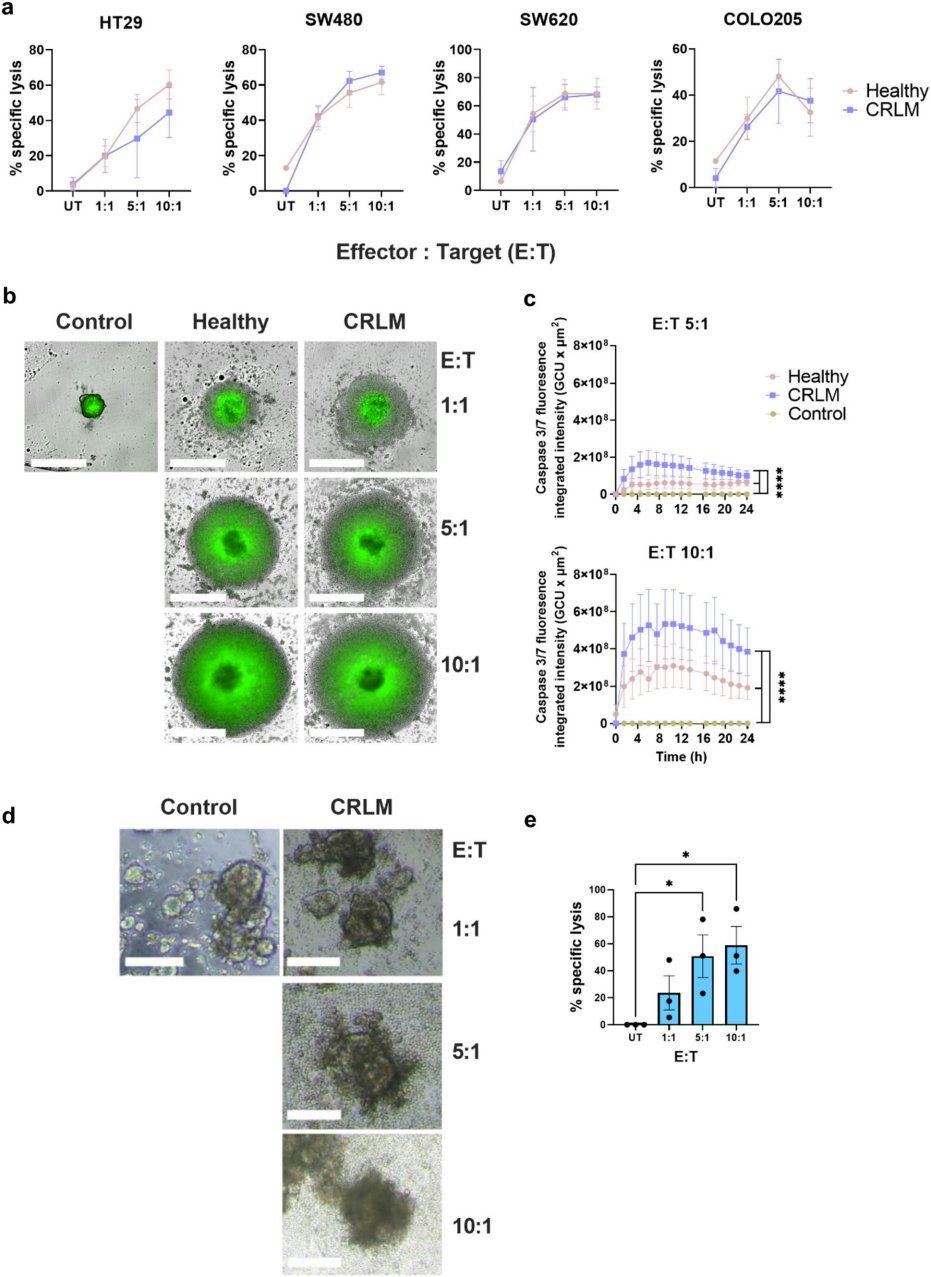
**Cytotoxicity of CRLM donor-derived CIK cells. a** 2D cytotoxicity assay with the HT29, COLO 205, SW480 or SW620 CRC cell lines as target cells and CIK cells at 10:1, 5:1, 1:1 effector to target (E:T) ratios. Target cells only were used as untreated (UT) controls. **b** 3D cytotoxicity assay with the HT-29 CRC cell line grown as spheroids as target cells and CIK cells at 10:1, 5:1, 1:1 E:T ratios, monitored over time using an Incucyte Live-Cell analysis system, **c** dynamic cleavage of caspase 3/7 (green) as a marker for cytotoxic activity measured over 24 h. White bars represent 100 µm. Caspase 3/7 graph shows mean ± SEM of triplicates. Data are representative of four independent experiments from separate donors. ****p < 0.0005. Two-way ANOVA with multiple comparison test were performed. **d** Autologous patient-derived tumour organoid (PDTO) 3D cytotoxicity assay (*n* = 3). Paired CRLM PDTO and CIK cell cytotoxicity assay from 3 independent donors. Representative images of CRLM PDTO (using PID-0169 as an example) disruption after co-culture with autologous CIK cells at different E:T ratios for 24 h. White bars represent 100 µm. **e** The absolute number of live CellTrace™ Violet labelled PDTOs was obtained by flow cytometry and % specific lysis was calculated in duplicate or triplicate. Results shown are mean ± SEM from three independent experiments derived from a unique donor each time. *p ≤ 0.05. One-way ANOVA with multiple comparison test were performed

Next, we compared the cytotoxic activity of CIK cells from CRLM and healthy donors using several cytotoxicity assays. In 2D cultures using CRC cell lines HT29, COLO 205, SW480 and SW620, there was comparable dose-dependent cytotoxicity between healthy and CRLM-derived CIK cells (Fig. 4a). There was also no difference between CIK cells in the induction of cell death in HT-29 spheroids (Fig. 4b–c). PDTOs are currently considered the best preclinical model to predict patient response to anti-cancer drugs and immunotherapy, including adoptive cell therapies [29, 30]. Using a well-described tumour organoid cytotoxicity assay [19], we confirmed that our CRLM donor-derived CIK cells can eliminate matched PDTOs (Fig. 4d–e). Together, these data indicate CIK cells generated from CRLM donors do not differ in in vitro cytotoxic capacity to CIK cells prepared from healthy donors and are able to kill tumour organoids.

### Effect of patient characteristics on CIK cell production

**Fig. 5:**
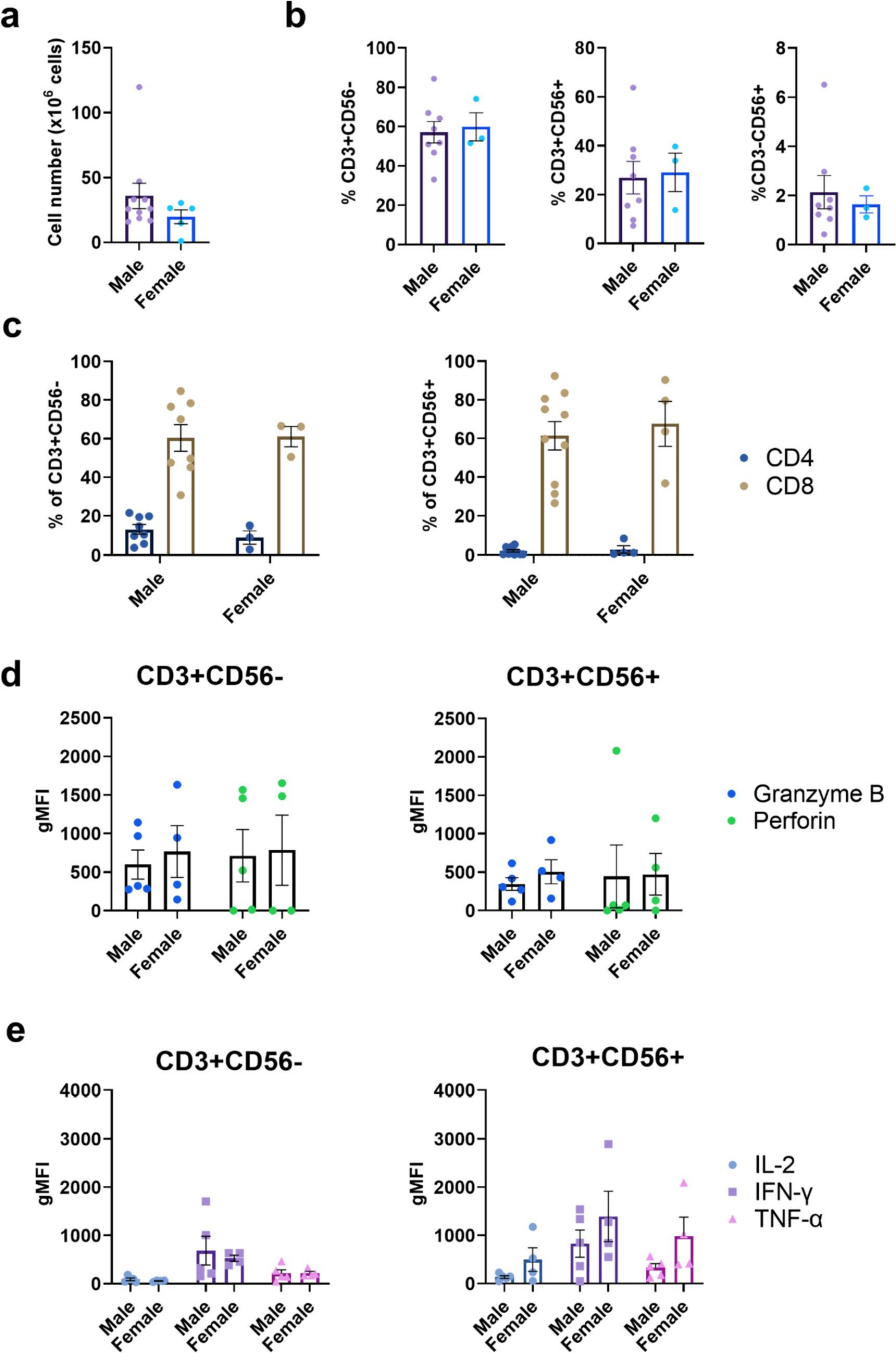
**The effect of biological sex on CIK cells. a** Total cell counts on day 21 post-culture for male and female healthy and CRLM donors. **b** Percentages of T (CD3 + CD56-), NK-like T cells (CD3 + CD56 +) and NK cells (CD3-CD56 +) in day 21 post-CIK cell culture. **c** The percentage of CD4 + and CD8 + subsets within the CD3 + CD56- and CD3 + CD56 + subpopulations at day 21 post-culture. **d** The gMFI for intracellular granzyme B and perforin and **e** IL-2, IFN-γ and TNF-α in CD8 + CD3 + CD56- and CD8 + CD3 + CD56 + CIK cells. Data are shown as mean ± SEM with each point representing an individual donor

Many studies have shown that biological sex has effects in cells of the adaptive immune response [31, 32]. We compared CIK cultures from females (*n* = 3) and males (*n* = 5) and found no significant difference in the number or percentage of CD3 + CD56-, CD3 + CD56 + , CD3-CD56 + subpopulations on day 21 post-culture (Fig. 5a, b). Sex did not affect the percentage of CD8 + CD3 + CD56- or CD8 + CD3 + CD56 + cells (Fig. 5c) nor did it influence the expression of granzyme B or perforin (Fig. 5d) or the production of type-1 cytokines (Fig. 5e).

**Fig. 6:**
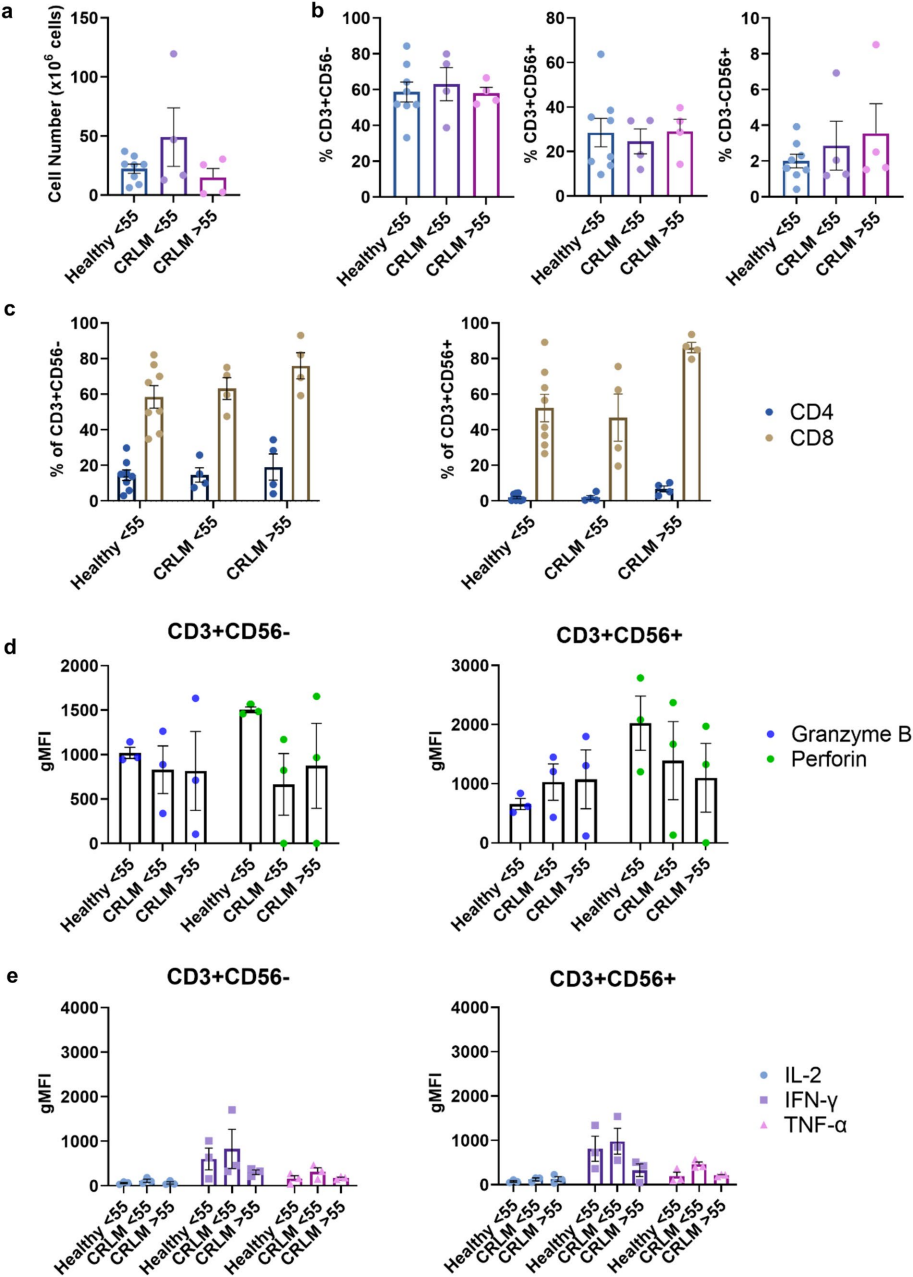
**The effect of age on CIK cells. a** Total cell counts on day 21 post-culture for different donor ages (Healthy < 55 years, CRLM < 55 years and CRLM > 55 years). **b** Percentages of T (CD3 + CD56-), NK-like T (CD3 + CD56 +) and NK cells (CD3-CD56 +) at day 21 post-culture. **c** Percentage of CD4 + and CD8 + subsets within CD3 + CD56- cells and CD3 + CD56 + subpopulations at day 21 post-culture. **d** The gMFI intracellular granzyme B and perforin and **e** IL-2, IFN-γ, TNF-α in CD8 + CD3 + CD56- and CD8 + CD3 + CD56 + CIK cells. Data are shown as mean ± SEM with each point representing an individual donor

Most patients with cancer tend to be older, and the immune system tends to deteriorate with age [33]. Since the current preference is to use autologous PBMC to generate CIK cells for patient use, it is important to know if age can affect the number or quality of CIK cells generated. We compared CIK cells generated from younger (< 55 years) and older (> 55 years) donors with CRLM and younger (< 55 years) healthy donors. We found no differences between these three groups in the total number of cells generated, the number or percentages of the subpopulations, or the expression of granzyme B or perforin, or the expression of the cytokines IL-2, IFN-γ or TNF-α (Fig. 6).

**Fig. 7:**
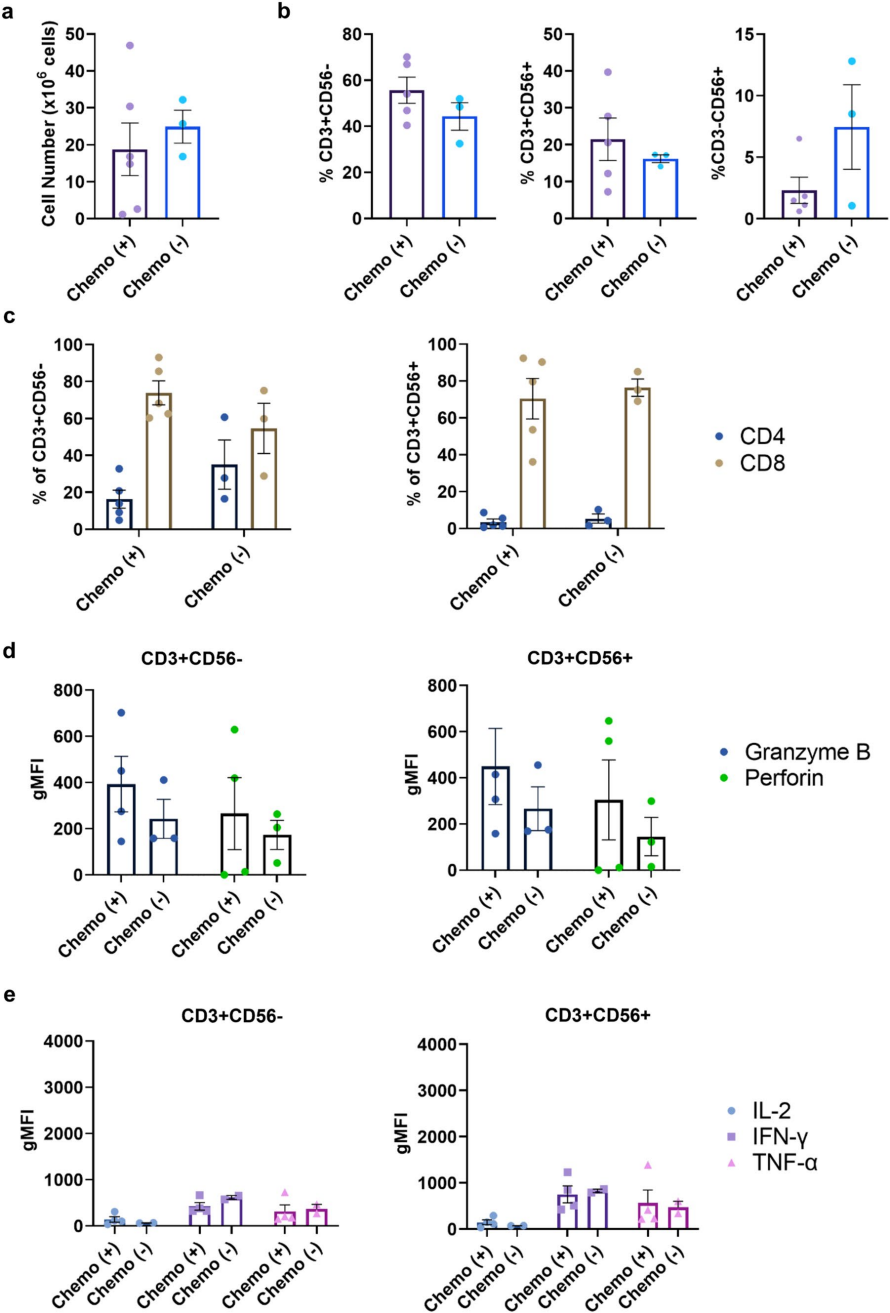
**The effect of chemotherapy exposure on CIK cells. a** Total cell counts on day 21 post-CIK cell culture for CRLM donors having received chemotherapy ( +) or not ( −) prior to PBMC collection. **b** Percentages of T (CD3 + CD56-), NK-like T (CD3 + CD56 +) and NK cells (CD3-CD56 +) at day 21 post-culture. **c** Percentage of CD4 + and CD8 + subsets within CD3 + CD56- cells and CD3 + CD56 + subpopulations at day 21 post-culture. **d** The gMFI intracellular granzyme B and perforin and **e** IL-2, IFN-γ, TNF-α in CD8 + CD3 + CD56- and CD8 + CD3 + CD56 + CIK cells. Data are shown as mean ± SEM with each point representing an individual CRLM donor

It has been suggested that recent chemotherapy treatment can have long-term effects on T cells that can impede the effectiveness of adoptive T cell therapies [34]. The impact of prior chemotherapy on autologous CIK cell generation is unknown. PBMCs from our patient cohort were collected just before curative-intent liver resection (Table 1). Importantly, a subset had received neoadjuvant chemotherapy prior to PBMC collection, which allowed us to address the influence of chemotherapy on CIK cell generation and function. We found no differences between chemotherapy naïve and treated patients in the total number of CIK cells generated, subset composition, or expression of the functional molecules granzyme B, perforin, IL-2, IFN-γ or TNF-α (Fig. 7). Taken together, we show that production of CIK cells is robust and highly feasible for a broad range of CRLM patients.

## Discussion

Despite the introduction of a number of new management strategies for patients with CRLM, 5-year survival remains poor and new treatments are required [35, 36]. Adoptive cell therapies, such as CIK cell therapy, are being actively investigated for treatment of a number of cancers. Reports that CIK cell therapy may result in significant clinical benefit in solid tumours, including CRC, suggest that its use should be considered more in western countries, at least for patients with CRC liver metastatic disease.

In the reported clinical studies, the CIK cell culture methods differ in detail and are often poorly described. For example, in four studies reporting CIK cell therapy for CRLM each used a different production protocol [14, 28, 37, 38]. Media used included serum free media or media supplemented with human serum or FBS ([11] and unpublished observations). While FBS supplementation is still common practice in cell culture for T cell therapies [39], its use increases the risk of xenoimmunisation and zoonotic disease transfer in recipients [16]. We found that X-VIVO 15, a GMP grade serum free medium, was comparable to serum supplemented RPMI in terms of CIK cell expansion, phenotypes, and cytotoxic capacities. Minor differences observed in X-VIVO 15 cultured CIK cells include lower levels of DNAM-1 and cytokine expression. These differences may be overcome by modifying the culture method, such as the addition of IL-15 [40]. Importantly, X-VIVO 15 and RPMI cultured CIK cells were comparably cytotoxic against CRC cells. Thus, X-VIVO 15 can be used as the base medium for large-scale CIK cell production for clinical trials.

In our hands long-term storage (6–12 months) of cryopreserved CIK cells reduced their cytotoxic capacity. Our cryopreservation method used FBS supplemented with 10% DMSO and was done in a small scale to suit laboratory investigation. It has been previously reported that supplementation with IL-2 after thawing may rejuvenate CIK cell functionality [41]. Two recent reports utilising clinical scale CIK cell cryopreservation techniques showed that long-term storage of CIK cells has minimal effects on their functionality [42, 43].

The numbers of CIK cells generated in culture, and their functional capacity, were similar whether the blood was from CRLM patients or healthy donors. These are important findings, given that it is preferable to use autologous rather than heterologous CIK cells for patient treatment. T cell fitness influences T cell manufacturing efficacy [44], and is reported to be impaired by cancer, and cancer treatment, as wells as chronic infection and ageing. Our findings show that CLRM patients do not have deficits in the mononuclear cells required to generate CIK cells in vitro. Within our limited sample size, we found that age, sex and prior chemotherapy exposure did not influence CIK cell production in CRLM patients. CIK cells were not generated from several blood samples. Whether this was due to a concurrent infection or other medical condition cannot be known as we did not have access to sufficient patient data. Currently, there is no consensus on how CIK cells are to be verified as functional prior to patient transfusion. This is the first report showing that autologous CIK cells are cytotoxic to matched PDTOs. Clinical trials which include PDTO cytotoxicity assays as pre-screens are required to determine if this predicts efficacy in vivo [45].

In conclusion, this is the first study to show that clinical grade CIK cells can be successfully generated in an Australian cohort of patients with CRLM. The production of CIK cells using a generic serum free CIK cell production protocol appears robust, with most patient factors not affecting the numbers or function of the CIK cells produced. Further investigation on the usage of CIK cell therapy for the treatment of CRLM in Australia is warranted.

### Supplementary Information

Below is the link to the electronic supplementary material.

Supplementary file1 (DOCX 3250 KB)

## Data Availability

All data generated or analysed during this study are included in this published article and its supplementary information files.

## Abbreviations

CAPOX: Capecitabine and oxaliplatin
ChRT: Concurrent chemoradiotherapy
CO_2_: Carbon dioxide
CIK: Cytokine-induced killer
CRC: Colorectal cancer
CRLM: Colorectal cancer liver metastases
DMEM: Dulbecco’s Modified Eagle Medium
DMSO: Dimethyl sulfoxide
DNAM-1: DNAX Accessory Molecule-1
DPBS: Dulbecco’s phosphate buffered saline
EDTA: Ethylenediaminetetraacetic acid
FACS: Fluorescence-activated cell sorting
FASL: Fas ligand
FBS: Foetal bovine serum
FOLFOX: Folinic acid, fluorouracil and oxaliplatin
GMP: Good manufacturing practice
h-EGF: Human epidermal growth factor
HEPES: N-2-hydroxyethylpiperazine-Nʹ-2-ethanesulfonic acid
IFN-γ: Interferon-gamma
IL-2: Interleukin-2
MHC: Major histocompatibility complex
N-acetyl-L cyst: N-acetyl-L-cysteine
NK: Natural killer
NKG2D: Natural-killer group 2 member D
PBMC: Peripheral blood mononuclear cell
PBS: Phosphate-buffered saline
PDTO: Patient-derived tumour organoid
Pen-Strep: Penicillin–streptomycin
Phenol-red: Phenolsulfonphthalein-red
RPMI: Roswell Park Memorial Institute
RT: Radiotherapy
SFM: Serum-free media
TNF-α: Tumour neurosis factor-alpha
TNT: Total-neoadjuvant therapy
TRAIL: TNF-related apoptosis-inducing ligand
